# One-step immortalization of primary human airway epithelial cells capable of oncogenic transformation

**DOI:** 10.1186/s13578-016-0122-6

**Published:** 2016-11-11

**Authors:** Jordan L. Smith, Liam C. Lee, Abigail Read, Qiuning Li, Bing Yu, Chih-Shia Lee, Ji Luo

**Affiliations:** 1 Laboratory of Cancer Biology and Genetics, Center for Cancer Research, National Cancer Institute, NIH., Bethesda, MD USA; 2 University of Massachusetts Medical School and the Graduate School of Biomedical Sciences, Worcester, MA USA; 3 Graduate Program, Cambridge University, Cambridge, UK; 4 ShanghaiTech University, Shanghai, China; 5 Janssen R&D Shanghai Discovery Center, Shanghai, China

## Abstract

**Background:**

The ability to transform normal human cells into cancer cells with the introduction of defined genetic alterations is a valuable method for understanding the mechanisms of oncogenesis. Easy establishment of immortalized but non-transformed human cells from various tissues would facilitate these genetic analyses.

**Results:**

We report here a simple, one-step immortalization method that involves retroviral vector mediated co-expression of the human telomerase protein and a shRNA targeting the *CDKN2A* gene locus. We demonstrate that this method could successfully immortalize human small airway epithelial cells while maintaining their chromosomal stability. We further showed that these cells retain p53 activity and can be transformed by the *KRAS* oncogene.

**Conclusions:**

Our method simplifies the immortalization process and is broadly applicable for establishing immortalized epithelial cell lines from primary human tissues for cancer research.

## Background

The evolution of cancer cells involves the acquisition of mutations that often fall within a set of defined genetic pathways. Experimental transformation of normal human cells into cancer cells through the introduction of defined oncogenic lesions represents a major breakthrough in cancer research, as this approach enables the step-by-step re-construction of the oncogenic process. It has been established that multiple alterations are required to transition primary human epithelial cells to a neoplastic/cancerous state in vitro. Primary human cells cultured in vitro experience two proliferation blockades, senescence and crisis [1]. When grown in chemically defined media without feeder cells, primary human epithelial cells undergo rapid senescence that is likely a result of cell culture stress [2]. Cell culture senescence is associated with the up-regulation of the tumor suppressor p16^INK4A^ and the subsequent activation of the Rb protein. Senescence can be bypassed with expression of viral oncoproteins that neutralize Rb and p53 activity [3–5]. These include the SV40 polyomavirus Large T (LgT) antigen [6, 7], the adenovirus E1A protein [1, 3, 5, 8], and the papillomavirus E6 and E7 proteins [2, 6, 9–11]. Human cells that have by-passed senescence still have limited replicative potential due to insufficient telomerase activity, and they eventually encounter crisis due to progressively shortening telomeres [1, 12, 13]. Re-expression of the catalytic subunit of telomerase, hTERT, which is sufficient to restore telomerase activity in many cell types [2, 14], can prevent telomere erosion, maintain genomic stability and immortalize cells [3–5, 15–18]. Historically, primary human cells have been immortalized through a two-step process: the first step involves the introduction of the aforementioned viral oncoproteins to neutralize Rb and p53 activity to bypass cell culture senescence [4, 6, 7]. The second step involves the introduction of hTERT, which serves to maintain telomere stability and prevent crisis [13]. Primary human epithelial cells immortalized this way can be successfully transformed by oncogenes such as Ras [19, 20]. This step-wise approach provides a valuable means to model malignant transformation under genetically defined conditions. Since most human cancer cells do not harbor viral oncoprotein expression, recent studies have sought to obviate the need for viral oncoproteins. It has been shown that the over-expression of the G1 cell cycle kinase CDK4 [21, 22] or shRNA-mediated knockdown of p16^INK4A^ [23, 24] can immortalize cells in the presence of hTERT.

Lung cancer is a leading cause of cancer-related mortality in the United States and worldwide. Approximately ~80% of lung cancer are non-small cell lung cancer (NSCLC) that is thought to originate from epithelial cells of the small airway or the alveolus [25–27]. Sequencing studies and copy number variation analyses have revealed that human lung adenocarcinomas frequently harbor mutations in *KRAS*, *TP53* and *CDKN2A* [28, 29]. Previously, several studies have showed that NSCLC can be modeled in vitro with human airway and bronchial epithelial cells [20, 30, 31]. In these studies, primary airway and bronchial epithelial cells were immortalized using hTERT together with either viral oncoproteins [30] or CDK4 overexpression [21]. Subsequent introduction of oncogenes such as *KRAS* could transform these cells and enable tumor growth in vivo [20, 30, 31].

Here we developed a simplified, one-step immortalization method for primary human cells and we demonstrated its utility in immortalizing human small airway epithelial cells (SAECs). We showed that immortalized SAECs are chromosomally stable and can be transformed by the *KRAS* oncogene in vitro. This approach should facilitate the establishment of isogenic panels of normal and transformed human cell lines for the study of malignant transformation.

## Results

### One-step immortalization of small airway epithelial cells

**Fig. 1:**
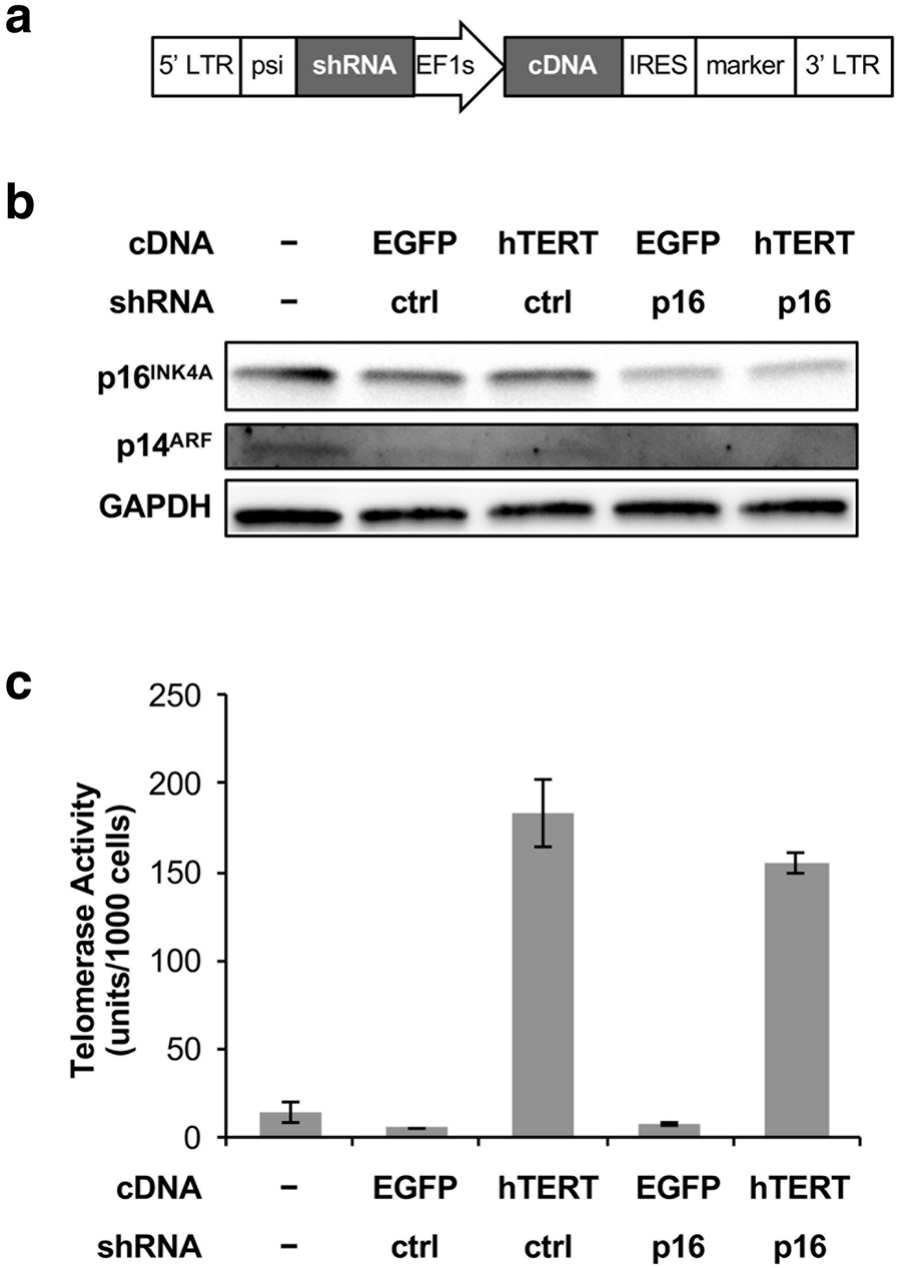
Characterization of retroviral vector for one-step immortalization. **a** Schematics of the MSCV-pic2 vector design. For one-step immortalization, a hTERT cDNA and a shRNA targeting p16^INK4A^ and p14^ARF^ were introduced into this vector. **b** Western blot verifying the knockdown of p16^INK4A^ and p14^ARF^ in stably transduced SAECs. **c** Telomerase activities in SAECs stably transduced with hTERT cDNA (*error bars* represent SD for all *bar charts* unless otherwise stated)

In the absence of feeder cells, the standard immortalization protocols for human epithelial cells typically involve a two-step process. First viral oncoproteins are introduced to bypass p16^INK4A^ and p53 dependent proliferation blockade induced by cell culture stress. Second, hTERT is re-expressed to prevent crisis and maintain genomic stability. To simplify the immortalization protocol and to avoid the use of viral oncoproteins, we designed a retroviral vector, MSCV-pic2, that is capable of co-expressing a shRNA and a cDNA on the same selectable marker (Fig. 1a). Using this vector, we simultaneously introduced into primary human SAECs a hTERT cDNA and a shRNA against the human *CDKN2A* locus that targets both the p16^INK4A^ and p14^ARF^ proteins (hereafter referred to as sh_p16). We reasoned that this should allow cells to proliferate both continuously while maintaining genomic stability in chemically defined media without feeder cells.

To validate our one-step immortalization approach, we engineered additional constructs that expressed a negative control shRNA targeting firefly luciferase (sh_ctrl) instead of sh_p16, and constructs that expressed the enhanced green fluorescent protein (EGFP) instead of hTERT. We transduced primary SAECs with these constructs and generated stable, polyclonal cell lines following drug selection. In the untransduced cells, p16^INK4A^ protein was expressed at moderate levels, whereas p14^ARF^ protein was expressed at very low levels (Fig. 1b, lane 1). Since our sh_p16 targets a common exon shared by both p16^INK4A^ and p14^ARF^, it knocked down both proteins by western blot (Fig. 1b, lanes 4, 5). To verify the activity of the hTERT cDNA, we used a PCR-based telomeric repeat amplification protocol (TRAP) assay to confirm that telomerase activity was indeed elevated in cells expressing hTERT but not in cell expressing EGFP (Fig. 1c).

**Fig. 2:**
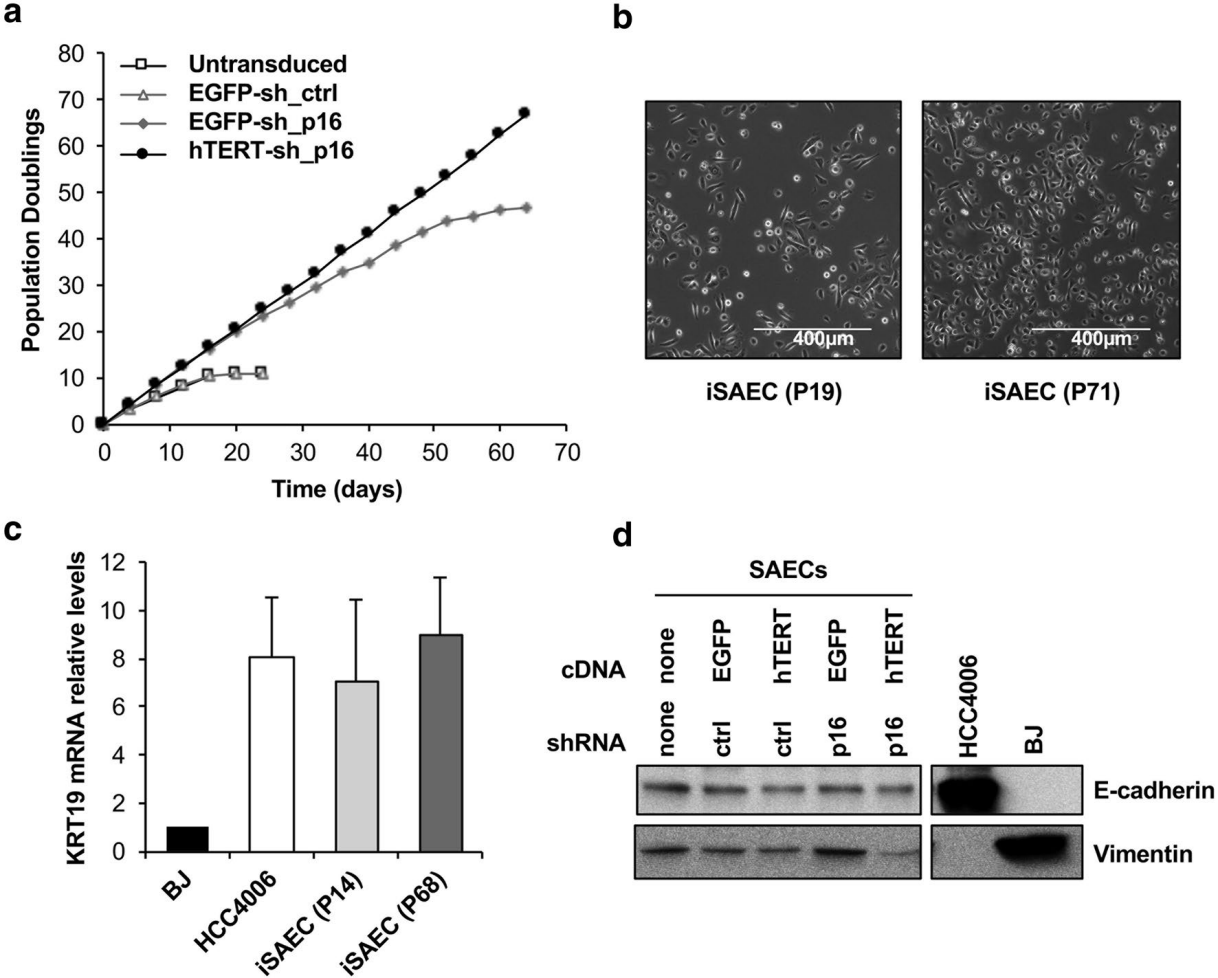
Characterization of the proliferation and morphology of iSAECs. **a** Population doubling (PD) curves for SAECs transduced with indicated cDNA and shRNA pairs. Only SAECs transduced with hTERT cDNA and sh_p16 were able to proliferate indefinitely. **b** Morphology of early and late passages (P19 and P71, respectively) iSAEC cells. **c** Expression of the epithelial marker keratin 19 (KRT19) in iSAECs. BJ fibroblasts and the NSCLC cell line HCC4006 were used as negative and positive controls, respectively. KRT19 mRNA levels were normalized to beta-actin. **d** Expression of E-cadherin and vimentin in iSAECs. BJ fibroblasts and the NSCLC cell line HCC4006 were used as controls

Next, we evaluated the proliferation capacity of SAECs stably expressing various combinations of hTERT and sh_p16. Similar to previous studies on airway epithelial cells [21], untransduced primary SAECs, as well as SAECs transduced with EGFP plus sh_ctrl only proliferated for a short period before senescing. SAECs expressing EGFP plus sh_p16, on the other hand, were able to bypass cell culture senescence and continue to proliferate for ~45 population doublings (PDs) before arresting, presumably as a result of reaching the Hayflick limit [12]. SAECs expressing hTERT plus sh_p16 were able to proliferate pass the Hayflick limit and maintain log phase proliferation beyond 70 PDs (Fig. 2a). Together, these results indicate that primary human epithelial cells can be successfully immortalized in a one-step process using our single vector system that that co-expresses hTERT and sh_p16. We thus referred to these immortalized SAECs as iSAECs.

### iSAEC morphology and genomic stability

Morphologically, iSAECs at early passage 19 (P19) and late passage (P71) exhibit similar features under phase-contrast microscopy (Fig. 2b). In contrast to BJ fibroblasts, iSAECs expressed the epithelial marker keratin-19, similar to the NSCLC cell line HCC4006 (Fig. 2c). Western blots showed that iSAECs expressed the epithelial marker E-cadherin, although they also expressed the mesenchymal marker vimentin at a low level (Fig. 2d). Thus we concluded that iSAECs have largely retained their epithelial characteristics throughout the immortalization process.

**Fig. 3:**
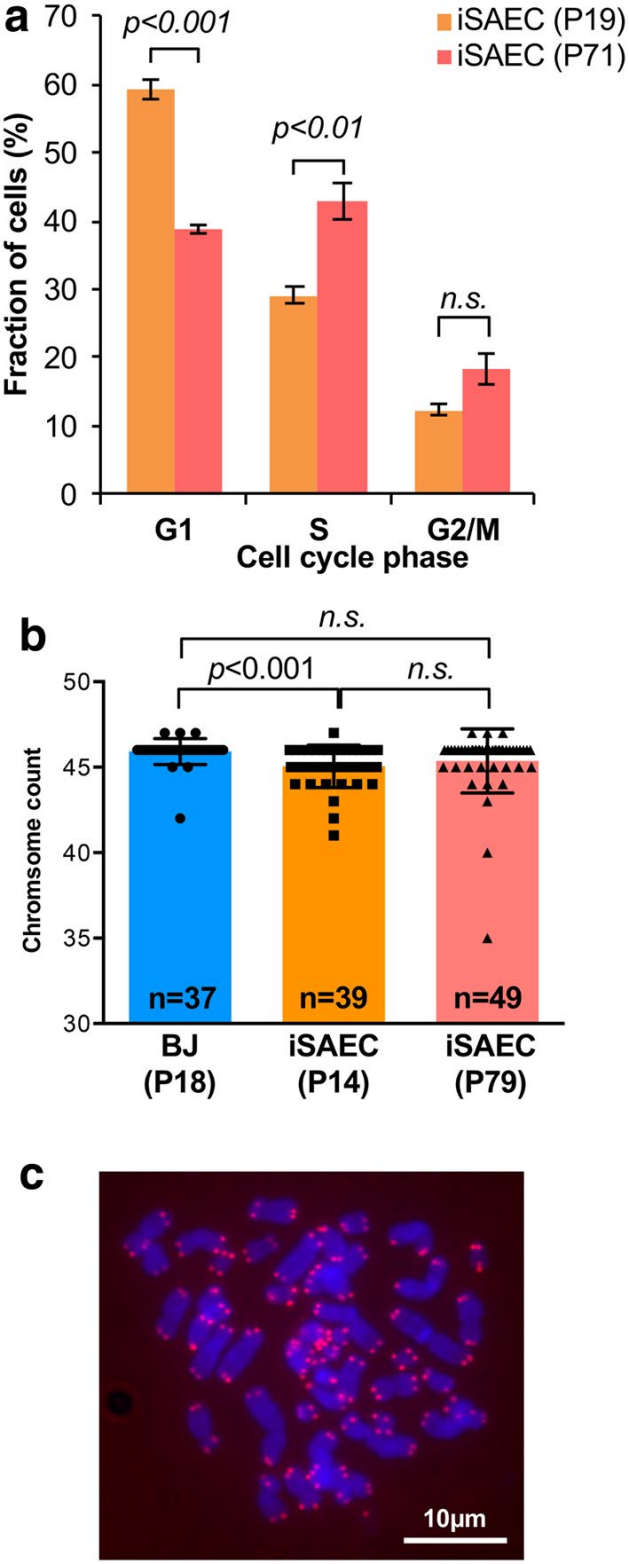
iSAECs are genomically stable. **a** Cell cycle distribution of early and late passage iSAECs in log phase culture (p values were determined using Student’s t test for all *bar charts*). **b** Chromosomal count of early and late passages (P14 and P79, respectively) iSAECs using metaphase spread of colcemid arrested mitotic cells. Early passage (P18) BJ fibroblasts were used as control. *Numbers* in each column indicates the number of metaphase spreads analyzed. **c** Telomere fluorescence in situ hybridization of late passage iSAECs. A representative metaphase spread image was shown

Next, we wanted to confirm that our genetic manipulation did not affect genomic stability in iSAECs. Cell cycle profiles of early passage (P19) and late passage (P71) SAECs showed that late passage cells have reduced G1 population and increased S phase population, although G2/M population remained similar (Fig. 3a). We evaluated the ploidy of iSAECs at early and late passages against early passage (P18) BJ fibroblasts using metaphase spreads. The majority of the iSAECs retained euploidy at both early and late passages (Fig. 3b), indicating that they were chromosomally stable. We noted that the early iSAECs tended to have higher fraction of hypo-diploid cells with 45 chromosomes, which might be a result of chromosome loss during sample preparation. Lastly, we stained late passage iSAECs for telomeres using PNA-Cy3 telomere probes. All chromosome ends stained positive for telomere and no chromosome fusions were observed (Fig. 3c). Together, these results indicate that iSAECs are chromosomally stable.

### Oncogenic transformation of iSAECs by mutant KRAS

**Fig. 4:**
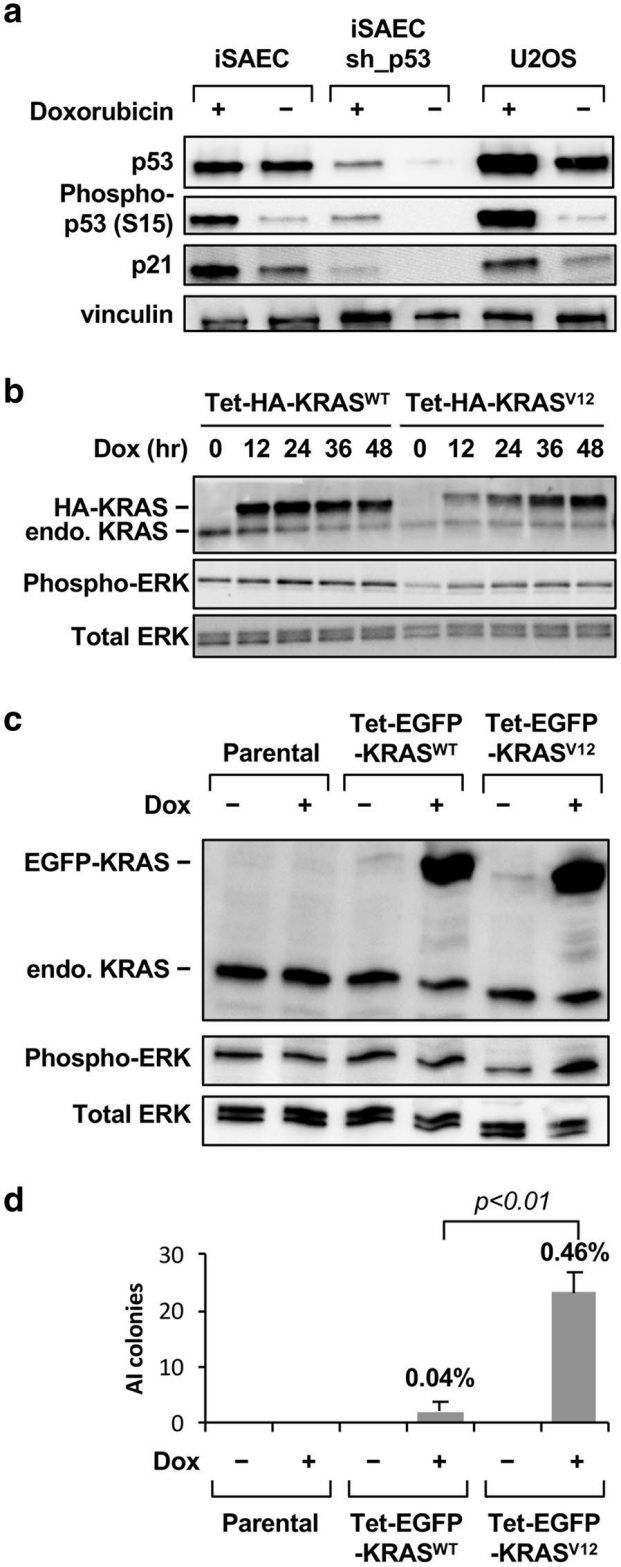
Response of iSAECs to p53 activation and KRAS oncogene transformation. **a** Induction of p53 by DNA damage in iSAECs. iSAECs with or without stable expression of a p53 shRNA (sh_p53) were treated for 24 h with 200 nM doxorubicin and phospho-p53 (Ser15) and p21 levels were measured by western blot. The p53 WT U2OS cells were included as a positive control. **b** Expression of tetracyclin (tet)-inducible HA-tagged KRAS^WT^ and KRAS^V12^ proteins in iSAECs. iSAECs stably expressing KRAS cDNA were treated with 100 ng/ml doxycycline for various periods of time and the level of KRAS and phospho-ERK (Thr202/T204) protein were measured. **c** Expression of tetracyclin (tet)-inducible EGFP-tagged KRAS^WT^ and KRAS^V12^ proteins in iSAECs. iSAECs stably expressing inducible KRAS cDNA were treated with 100 ng/ml doxycycline for 2 days and the level of KRAS and phospho-ERK protein were measured. **d** Soft agarose anchorage-independent (AI) colony assay of iSAECs cells following induction of EGFP-KRAS^WT^ and EGFP-KRAS^V12^ proteins. 5000 iSAECs were seeded in soft-agarose media with or without 100 ng/ml doxycycline and the colony numbers were counted 16 days later. The *percentage values* above each bar graph indicate clonogenic efficiency as a % of the total number of cells seeded

We designed our immortalization system with the goal of keeping some degree of intact p53 response. Because the sh_p16 shRNA targets both the p16^INK4^ and p14^ARF^ proteins, we recognize that the p14^ARF^-mediated regulation of p53 function is likely to be affected. However, because in SAECs p14^ARF^ level was very low to begin with (Fig. 1b), we reason that p53 response should at least be partially preserved in iSAECs. Indeed, p53 protein was readily detectable in iSAECs when compared to the osteosarcoma cell line U2OS which retains WT p53 expression (Fig. 4a). Furthermore, when iSAECs were treated with the DNA damage agent doxorubicin, both p53 phosphorylation and the induction of the p53 target protein p21 were robustly induced in iSAECs (Fig. 4a). Of note and different from U2Os cells, doxorubicin treatment did not substantially increase total p53 protein level in iSAECs. In addition, when we introduced a p53 shRNA (sh_p53) into iSAECs, we were able to deplete p53 and ablate doxorubicin-induced p21 induction (Fig. 4a). Thus, we concluded that iSAECs retain p53 function and this allows the independent manipulation of p53 activities in these cells.

Lastly, we wished to determine how iSAECs would respond to oncogenic transformation by the *KRAS* oncogene. We generated iSAECs stably expressing HA-tagged WT KRAS protein (KRAS^WT^) or the constitutively active mutant KRAS-G12V protein (KRAS^V12^) under the control of a tetracycline (tet)-inducible promoter. In these cells, doxycycline treatment leads to a dose-dependent induction of HA-KRAS (Fig. 4b). Unexpectedly, induction of KRAS^V12^ did not dramatically activate the MAPK pathway as measured by ERK kinase phosphorylation (Fig. 4b). Neither was this level of KRAS^V12^ expression able to confer anchorage-independent grow in soft agarose. We next used a tet-inducible EGFP-KRAS^V12^ fusion construct which can be expressed at a higher level to elevate phospho-ERK level (Fig. 4c). Under these conditions the mutant EGFP-KRAS^V12^ protein enabled AI colony formation (Fig. 4d). However, the clonogenic efficiency was low and only ~0.5% of cells formed soft agarose colonies. Thus, iSAECs are capable of being transformed by the *KRAS* oncogene at a low efficiency, and it is likely that additional genetic perturbations are required for their full transformation [31].

## Discussion

In this study, we developed a method that enables the one-step immortalization of human primary epithelial cells through the simultaneous introduction of hTERT cDNA and a shRNA against the *CDKN2A* locus. We demonstrate that this approach led to the establishment of immortalized cells capable of replication beyond the Hayflick limit [12] while maintaining telomeres and genomic stability.

We avoided using viral oncoproteins such as LgT, E6 and E7 in our approach as these proteins inactivate both the Rb and p53 pathway. We demonstrate that p16^INK4A^ knockdown in combination with hTERT was sufficient for cell immortalization, similar to a previous report [24]. Due to the shared exon usage between p16^INK4A^ and p14^ARF^ proteins, we were unable to identify a potent shRNA that selectively knockdown p16^INK4A^ but not p14^ARF^. However, in the case of SAECs, p14^ARF^ expression level was very low and its depletion did not lead to loss of p53 proteins. Indeed, we found that in proliferating iSAECs p53 was expressed and could be readily activated by DNA damage. Thus, p53 function is at least partially preserved in iSAECs and full p53 inactivation may not be an obligatory requirement for the immortalization of these cells [21, 24]. Interestingly, in iSAECs, total p53 protein level was not substantially elevated by DNA damage. This was unexpected as phosphorylation typically stabilizes p53 by interfering with its binding to the E3 ligase MDM2 [32]. Whether this phenomenon is specific to SAECs or is associated with our particular method of cell immortalization require further investigation. Nevertheless, our approach allows p53 activity to be manipulated separately, as we demonstrated with shRNA mediated p53 knockdown. This is a useful feature as it enables the study of genetic interaction between p53 and other oncogene and tumor suppressors in this model system.

In iSAECs, the introduction of the *KRAS* oncogene could drive soft-agarose colony growth. However, the transformation efficiency was low and it required relatively high levels of KRAS expression. Our findings thus indicate that human airway epithelial cells are relatively resistant to malignant transformation elicited by a single oncogene. This is in agreement with previous studies indicating that KRAS alone was unable to fully transform immortalized airway epithelial cells and cooperation from additional oncogenes including *PIK3CA* and *MYC* are necessary for their full transformation [20, 22, 31].

A convenient method to immortalize cells serves two valuable purposes. First, immortalized cells such as iSAECs provide a starting point for the evaluation of oncogene and tumor suppressor function in a prospective, isogenic setting. This enables step-wise reconstruction of the oncogenic process by the introduction of defined genetic changes [19, 20, 22, 30, 31]. Second, immortalized but non-transformed epithelial cells are valuable “normal” controls for tumor cell lines for evaluating drug target and drug candidate toxicity. Currently, many human cancer cell lines lack “normal” counterparts from the same tissue. Although we only tested SAECs in this study, it is likely that, with the appropriate media conditions, our approach should facilitate the creation of immortalized epithelial cell lines from various tissues for cancer research.

## Methods

### Cell culture and pharmacological agents

Primary human small airway epithelial cells were purchased from Lonza. Cells were cultured in SAGM growth media (Lonza). BJ Fibroblasts (ATCC) were cultured in eagle’s minimum essential medium (ATCC) supplemented with 10% heat inactivated fetal bovine serum (Hi-FBS, Life Technologies) and 100 units/mL penicillin plus 100 µg/ml streptomycin (P/S, Lonza). U2OS cells were cultured in McCoy’s 5A Media (Lonza) supplemented with 10% Hi-FBS and P/S. Cells were maintained at 37 °C in 5% CO_2_. Doxorubicin was used at a concentration of 200 nM for cell culture treatment. KaryoMAX^®^ Colcemid™ Solution in PBS (Life Technologies) was used at a concentration of 0.1 ug/mL for metaphase spreads. Doxycycline was from Sigma.

### Plasmid construction and generation of stable cell lines

MSCV-pic2 is a retroviral vector that co-expresses a cDNA and a shRNA. For one-step immortalization, we introduced into this vector the cDNA of the catalytic subunit of hTERT and a shRNA for the *CDKN2A* gene locus that knocks down both p16^INK4A^ and p14^ARF^. Plasmids generated using the MSCV-pic2 vectors include: MSCV-pic2-neo-hTERT-sh_p16, MSCV-pic2-neo-EGFP-sh_p16, MSCV-pic2-neo-hTERT-sh_Ctrl, MSCV-pic2-neo-EGFP-sh_Ctrl. The shRNA target sequences were: sh_p16 (ACTCGGGAAACTTAGATCATCA), sh_p53 (CCCGGCGCACAGAGGAAGAGAA), sh_Ctrl (firefly luciferase shRNA CCCGCCTGAAGTCTCTGATTAA). The lentiviral tet-inducible vectors expressing EGFP-KRAS V12 and WT proteins were described before [33]. Plasmids were packaged in 293T cells with *Trans*-IT-293 Transfection Reagent (Mirus Bio). SAECs were transduced by spin infection at 1800 RPM for 45 min. Stable cell lines were generated using the drug selectable markers associated with each vector.

### Western blots

Cells were lysed directly with Laemmli sample buffer. Whole cell lysates were boiled for 10 min at 95 °C and subsequently stored at −80 °C. Whole cell lysates were separated using BioRad Mini-Protean TGX 4–20% resolving gels. The protein was then transferred to a nitrocellulose membrane. The source of antibodies were: p16^INK4A^ (BD Bioscience #551153), p14^ARF^ (Bethyl, A300-340A), p53 (Santa Cruz #DO-1 SC-126), p21 (Calbiochem #OP64) Vinculin (Sigma #V9131), GAPDH (Santa Cruz #SC-25778) and phospho-p53-S15 (Cell Signaling Technology #9284), KRAS (Sigma, clone 4F3), phospho-ERK (Cell Signaling Technology, #4377), total ERK (Cell Signaling Technology, #9102), phospho-Akt (Cell Signaling Technology, #4058), Akt (Cell Signaling Technology, #9272), E-Cadherin (Cell Signaling Technology, #24E10), N-Cadherin (Cell Signaling Technology, #13116), Vimentin (Cell Signaling Technology, #5741). Blots were developed using conjugated anti-rabbit or anti-mouse and either Luminata Forte (Millipore) substrate or SuperSignal West Femto (Pierce) substrate on an Alpha Innotech HD2 Western Blot Imaging Station (protein Simple). Images were quantified with Alpha Innotech Image Software or Adobe Photoshop. Cropping and contrast adjustment were applied to entire images consistently without local alterations.

### Quantitative reverse transcription PCR

RNA was extracted from early and late passage iSAECs, BJ Fibroblasts, and HCC4006 NSCLC cell line using the RNA easy Kit and QiaShredder Columns (Qiagen). Collected RNA was reverse transcribed to cDNA using Multiscribe Reverse Transcriptase (Applied Biosystems). Real-time PCR was performed on the ABI 79300HT Real-Time Thermo Cycler with SYBR Green using the following primers pairs: KRT19_F (ACCAAGTTTGAGACGGAACAG) and KRT19_R (CCCTCAGCGTACTGATTTCC), B-Actin_F (AGAGCTACGAGCTGCCTGAC) and B-Actin_R (AGCACTGTGTTGGCGTACAG). All mRNA levels were normalized to B-Actin levels and KRT19 levels in BJ Foreskin Fibroblasts. mRNA levels are the average of three technical replicates, and error bars are standard deviation.

### Cell cycle analysis flow sorting

Log phase iSAEC passage 19 (P19) and iSAEC (P71) were collected, stained and fixed with propidium iodide (Sigma-Aldrich). Cells were scanned with a FACS Calibur analyzer (Beckson-Dickinson) and the data acquired with CellQuest Pro software. FACS profiles were analyzed using ModFit LT for all samples. The experiment was performed with three biological replicates of iSAECs.

### Metaphase spreads, FISH, and microscopy

Log-phase, iSAEC (P14), iSAEC (P79) and BJ Fibroblasts (P18) were treated with KaryoMAX^®^ Colcemid™ Solution in PBS at 0.1 ug/mL for 6 h at 37 °C. Mitotic fractions of cells were collected and pre-warmed 75 mM hypotonic solution was added to the mixture and incubated at 37 °C for 25 min. Mitotic cells were fixed in a solution of 3:1 v/v Methanol to Acetic Acid. Slides were prepared and stained with SlowFade^®^ Gold Antifade Mountant with DAPI (Life Technologies). Metaphase spreads were imaged with Zeiss Axio Microscope on a 63× Oil Objective. Metaphase spreads were quantified manually using Adobe Photoshop for labeling and isolation of chromosomes. Metaphase spreads of iSAEC (P61) were prepared as described above. Before the addition of DAPI, slides were stained with Telomere PNA Cy3 probe (DAKO) according to the manufacturer’s instructions and then cross-stained with SlowFade^®^ Gold Antifade Mountant with DAPI. Telomere probe slides were imaged with Zeiss Axio Imager on a 63× Oil Objective.

### Telomerase assay

Telomerase activity was quantified using the TRAPEZE RT Telomerase Detection Kit (Millipore) according to the manufacturer’s instructions.

### Anchorage independent growth assays

Cells were plated at 5000 cells/well in 6-well plates in triplicates in soft-agarose. For tet-inducible EGFP-KRAS expression, doxycycline (100 ng/ml) was included in both the agarose-media mix. Cells were allowed to grow for 16 days. Colonies were stained with 0.005% crystal violet in 5:4:1 methanol: water: acetic acid. Colonies were quantified using the Alpha Innotech Imaging Station Colony Counter Software.

### Population doubling

Stably transduced SAEC-hTERT-sh_p16, SAEC-GFP-sh_p16, SAEC-GFP-FF2, and untransduced SAECs were seeded in a 6-well plate and continuously passaged in log-phase for approximately 90 days. Media was changed every other day, and population doubling was measured every three to four days when confluency reached 80–90%.

## Abbreviations

AI: anchorage-independent
EGFP: enhanced green fluorescent protein
LgT: Large-T
NSCLC: non-small cell lung cancer
SAECs: small airway epithelial cells
tet: tetracycline
TRAP: telomeric repeat amplification protocol
